# Ethylene-*alt*-α-Olefin Copolymers by Hydrogenation of Highly Stereoregular *cis*-1,4 Polydienes: Synthesis and Structural Characterization

**DOI:** 10.3390/molecules29061376

**Published:** 2024-03-20

**Authors:** Giovanni Ricci, Antonella Caterina Boccia, Ivana Pierro, Claudio De Rosa, Miriam Scoti

**Affiliations:** 1 Consiglio Nazionale delle Ricerche (CNR)—Istituto di Scienze e Tecnologie Chimiche “Giulio Natta” (SCITEC), Via A. Corti 12, I-20133 Milano, Italy; antonella.boccia@scitec.cnr.it; 2 Scientific Advisor, I-21052 Busto Arsizio, Italy; ivanapierro@gmail.com; 3 Dipartimento di Scienze Chimiche, Università di Napoli Federico II, Complesso Monte S. Angelo, Via Cintia, I-80126 Napoli, Italy; claudio.derosa@unina.it (C.D.R.); miriam.scoti@unina.it (M.S.)

**Keywords:** polymers hydrogenation, olefin copolymers, polymer characterization

## Abstract

The homogeneous non-catalytic hydrogenation of several types of iso- and syndiotactic *cis*-1,4 poly(1,3-diene)s with diimide, formed by thermal decomposition of *p*-toluene-sulfonyl-hydrazide, was examined. Perfectly alternating ethylene/1-alkene copolymers having different tacticity (i.e., isotactic and syndiotactic), which in some cases are difficult to synthesize by simple stereospecific co-polymerization of the corresponding monomers, were obtained. All the copolymers synthesized were fully characterized from a structural, morphological, and rheological point of view through different analytical techniques (FT-IR, NMR, GPC, DSC, RX).

## 1. Introduction

**Scheme 1:**
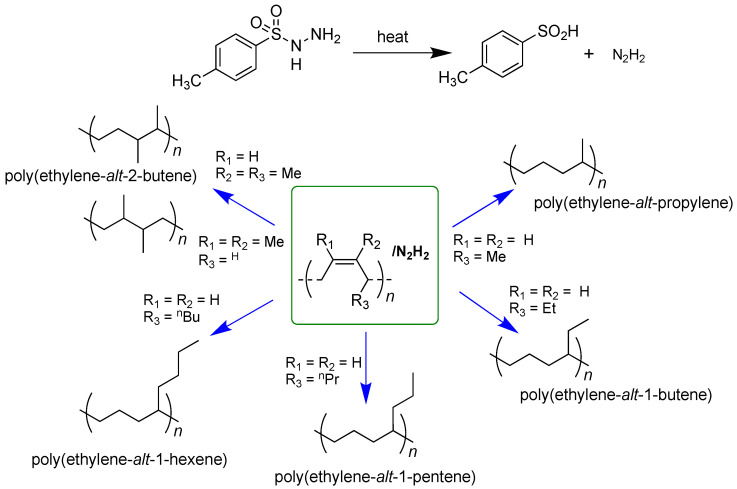
Perfectly alternating isotactic and syndiotactic ethylene/*a*-olefin copolymers by hydrogenation of highly stereoregular iso- and syndiotactic *cis*-1,4 polydienes.

The stereospecific polymerization of 1,3-dienes with transition metal and lanthanide catalysts can give polymers with different structures (*cis*-1,4; *trans*-1,4; 1,2; 3,4; iso-and syndiotactic) from different monomers [1,2,3]. The polymer microstructure mainly depends on the catalyst and monomer structures and particularly on the combination of these two factors [4,5,6]. Thus for instance, the classical ternary system AlEt_2_Cl/Nd(OCOC_7_H_15_)_3_/Al(*i*Bu)_3_ [7,8] gives isotactic *cis*-1,4 polymers from various substituted butadienes such as (*E*)-1,3-pentadiene [9], (*E*)-3-methyl-1,3-pentadiene [10,11], 1,3-hexadiene [12], 1,3-heptadiene [13] and 1,3-octadiene [13], while some nickel and cobalt catalysts (e.g., Ni(acac)_2_-MAO; Co(acac)_3_-MAO; CoCl_2_(P^t^Bu_2_Me)_2_-MAO) give highly syndiotactic *cis*-1,4 polymers from the same monomers [13,14,15]. The great availability of all these highly stereoregular polydienes may represent an outstanding source of highly stereoregular olefin copolymers, hardly obtainable through the simple and common stereospecific polymerization of the corresponding monomers. In fact, the hydrogenation of isotactic and syndiotactic *cis*-1,4 polydienes can give perfectly alternating isotactic and syndiotactic ethylene/α-olefin copolymers, as shown in the following Scheme 1.

We have already reported on the synthesis and characterization of isotactic and syndiotactic perfectly alternating ethylene/2-butene [16] and ethylene/propylene [17] copolymers.

In the present paper we are now describing the synthesis and characterization of (i) perfectly alternating isotactic ethylene/1-butene copolymers (E/1-B), obtained by hydrogenation of isotactic *cis*-1,4 poly(1,3-hexadiene) [12]; (ii) perfectly alternating ethylene/1-pentene copolymers (E/1-P), iso- and syndiotactic, obtained by hydrogenation of *cis*-1,4 poly(1,3-heptadiene)s, iso- and syndiotactic [13]; (iii) perfectly alternating ethylene/1-hexene copolymers (E/1-H), iso- and syndiotactic, obtained by hydrogenation of *cis*-1,4 poly(1,3-octadiene)s, iso- and syndiotactic [13]. 

A wide variety of catalysts and comonomers has been investigated for the ethylene/α-olefin copolymerization [18,19,20,21,22,23,24,25,26,27,28,29,30,31,32,33,34]. Alternating or pseudo-alternating ethylene/α-olefin copolymers were obtained up to now only with metallocene-based catalysts, which, with respect to the classical heterogeneous ZN (Ziegler-Natta) catalysts, exhibit much smaller r_e_ values and larger r_α-olefin_ values, allowing indeed for a more uniform comonomer distribution. The hydrogenation of highly stereoregular polydienes may represent a valid alternative to the above processes.

## 2. Results

As mentioned above, the highly stereoregular *cis*-1,4, iso- and syndiotactic, polydienes on which this study focused were the following: isotactic *cis*-1,4 poly(1,3-hexadiene) [hereafter named (*cis*1,4^iso^PHX)], syndiotactic *cis*-1,4 poly(1,3-heptadiene) (*cis*1,4^sy^PHP), isotactic *cis*-1,4 poly(1,3-heptadiene) (*cis*1,4^iso^PHP), syndiotactic *cis*-1,4 poly(1,3-octadiene) (*cis*1,4^sy^PO), and isotactic *cis*-1,4 poly(1,3-octadiene) (*cis*1,4^iso^PO). The syndiotactic *cis*-1,4 polymers were synthesized with the catalyst system CoCl_2_(P^t^BuMe_2_)_2_/MAO (MAO = methylalumoxane) [13]; the isotactic *cis*-1,4 polymers were obtained with the ternary catalyst system AlEt_2_Cl/Nd(OCOC_7_H_15_)_3_/Al^i^Bu_3_ [10,12,13]. The polymerization data concerning the preparation of all the diene-polymers are summarized in Table 1.

The hydrogenation of the above polymers was then examined. The hydrogenation is a type of chemical modification of polymers, allowing to reduce the amount of unsaturation, and leading to polymers with modified and improved properties and to the production of new materials [28]. Nevertheless, the hydrogenation may also be seen as a possible route to provide polymers which can be hardly prepared by a simple conventional polymerization of the corresponding monomers.

There are different methods to hydrogenate poly(1,3-diene)s, which involve catalytic and non-catalytic processes. The hydrogenation of unsaturated polymers in a non-catalytic way, in which the reaction is promoted by diimide (diazene, NH=NH), turned out to be an attractive process and extremely efficient in the case of 1,3-diene polymers [28,29,35,36,37].

Diimide hydrogenation is a non-catalytic process in which the hydrogenated polymers are formed through the reduction reaction between the diimide and the olefinic group (-C=C-). The diimide molecule (N_2_H_2_), as the hydrogenation agent, is generated in situ from the thermal decomposition of *p*-toluenesulfonyl hydrazide (TSH). The diimide molecule (N_2_H_2_) can then release a hydrogen molecule directly to the carbon-carbon double bonds, thus allowing the hydrogenation process.

In this work, we exploited the non-catalytic hydrogenation with diimide in homogeneous conditions at 120 °C, with *o*-xylene as solvent, of the following highly stereoregular polydienes: isotactic *cis*-1,4 poly(1,3-hexadiene), isotactic *cis*-1,4 poly(1,3-heptadiene), isotactic *cis*-1,4 poly(1,3-octadiene), syndiotactic *cis*-1,4 poly(1,3-heptadiene), syndiotactic *cis*-1,4 poly(1,3-octadiene). The hydrogenation process converted the *cis*-1,4 poly(1,3-hexadiene), poly(1,3-heptadiene)s, and poly(1,3-octadiene)s into perfectly alternating ethylene/1-butene, ethylene/1-pentene and ethylene/1-hexene copolymers (Scheme 1), having iso- or syndiotactic structure as a function of the starting diene polymer, respectively. 

Hereafter we will call H(*cis*1,4^iso^PHX) the alternating E/1-B copolymers arising from the hydrogenation of isotactic *cis*-1,4 poly(1,3-hexadiene), H(*cis*1,4^iso^PHP) and H(*cis*1,4^sy^PHP) the alternating E/1-P copolymers arising from the hydrogenation of isotactic and syndiotactic *cis*-1,4 poly(1,3-heptadiene), H(*cis*1,4^iso^PO) and H(*cis*1,4^sy^PO) the alternating E/1-H copolymers arising from the hydrogenation of isotactic and syndiotactic *cis*-1,4 poly(1,3-octadiene). 

Data concerning tacticity, molecular weight and molecular weight distribution, and glass transition temperature for all the hydrogenated polymers, are summarized in Table 2.

### 2.1. Isotactic Alternating Ethylene/1-Butene Copolymer (H(cis1,4^iso^PHX))

**Scheme 2:**
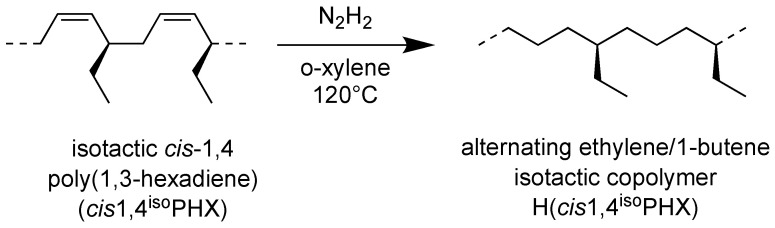
Scheme of hydrogenation of isotactic *cis*-1,4 poly(1,3-hexadiene) (*cis*1,4^iso^PHX) to isotactic ethylene-alt-1-butene copolymer H(*cis*1,4^iso^PHX).

The hydrogenation process of the isotactic *cis*-1,4 poly(1,3-hexadiene) was illustrated in Scheme 2. 

**Figure 1:**
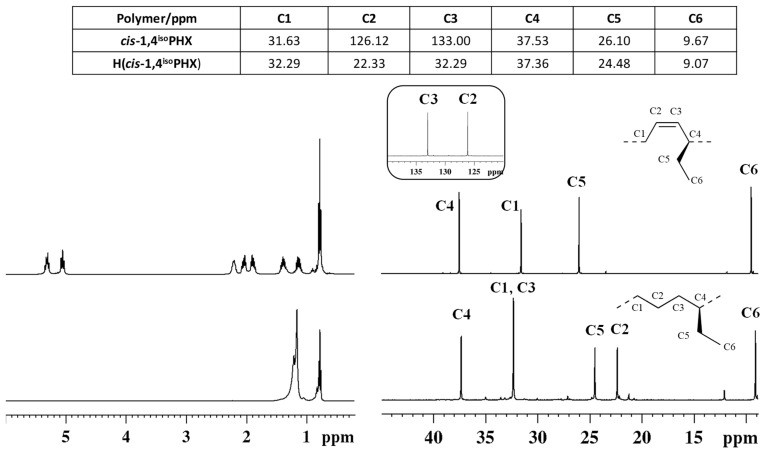
^1^H (**left**) and ^13^C NMR (**right**) spectra of isotactic *cis*-1,4 poly(1,3-hexadiene) ((**top**), (*cis*1,4^iso^PHX)) and its saturated E/1-B copolymer ((**bottom**), H(*cis*1,4^iso^PHX)).

The successful complete hydrogenation of the diene polymer was confirmed by comparison of the Fourier transform infrared (FT-IR) (Figure S1) and ^1^H and ^13^C NMR spectra (Figure 1) of the starting *cis*-1,4 poly(1,3-diene) and of the corresponding hydrogenated product.

The typical band observed at 751 cm^−1^ in the FTIR spectrum of *cis*-1,4 poly(1,3-hexadiene), ascribed to the out-of-plane vibration of the hydrogen atoms adjacent to the double bond in a *cis*-1,4 unit, is completely absent in the FT-IR spectrum of the corresponding hydrogenated polymer; besides, a new band at 735 cm^−1^ is observed in the FT-IR spectrum of the hydrogenated polymer, ascribed to the vibration of a –CH_2_– unit, typical of saturated polyolefins (Figure S1)

The ^1^H and ^13^C NMR spectra of the isotactic *cis*-1,4 poly(1,3-hexadiene) and of the corresponding hydrogenated product are shown in Figure 1. As it is clearly evident, peaks in the olefinic region observed in the ^1^H NMR (from 5.2 to 5.4 ppm) and ^13^C NMR (from 120 to 140 ppm) spectra of the diene polymers, and due to the olefinic hydrogen and carbon atoms, are not observed in both the NMR spectra of the hydrogenated polymer, confirming indeed the complete hydrogenation of the diene polymer. 

The structure of the ethylene /1-butene copolymer in Figure 1 was determined by means of ^1^H, ^13^C, and two-dimensional NMR experiments, such as ^1^H-^13^C heteronuclear experiments (HSQC and HMBC in Figure S3) and two-dimensional ^1^H-^1^H homonuclear experiments (COSY and TOCSY Figure S4).

**Figure 2:**
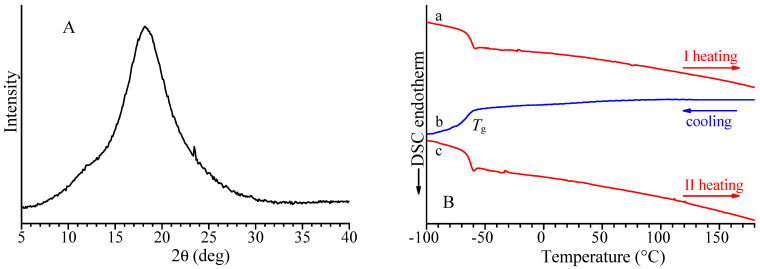
X-ray powder diffraction profile (**A**) and DSC curves (**B**) recorded at 10 °C/min during first heating (a), successive cooling (b), and second heating (c) of alternating E/1-B copolymer (sample H(*cis*1,4^iso^PHX)).

The X-ray diffraction profile of the alternating isotactic ethylene/1-butene copolymer is shown in Figure 2A, and the DSC curves recorded during the first heating, successive cooling from the melt, and second heating of the melt crystallized samples are reported in Figure 2B. Both X-ray diffraction and DSC data indicate the absence of crystallinity in this sample. The alternating isotactic ethylene/1-butene copolymer does not crystallize after annealing or cooling from high temperatures, as confirmed by the absence of exothermic peaks or endothermic peaks in the DSC cooling and heating curves. The DSC shows only a glass transition temperature of −66 °C (Figure 2B and Table 2). As in the case of alternating stereoregular ethylene/propene copolymers prepared from hydrogenation of isotactic and syndiotactic *cis*-1,4 poly(1,3-pentadiene) [17], the alternating isotactic ethylene/1-butene copolymer is not able to crystallize notwithstanding the regular stereochemical structure.

### 2.2. Iso- and Syndiotactic Alternating Ethylene/1-Pentene Copolymers (H(cis1,4^iso^PHP) and H(cis1,4^sy^PHP))

**Scheme 3:**
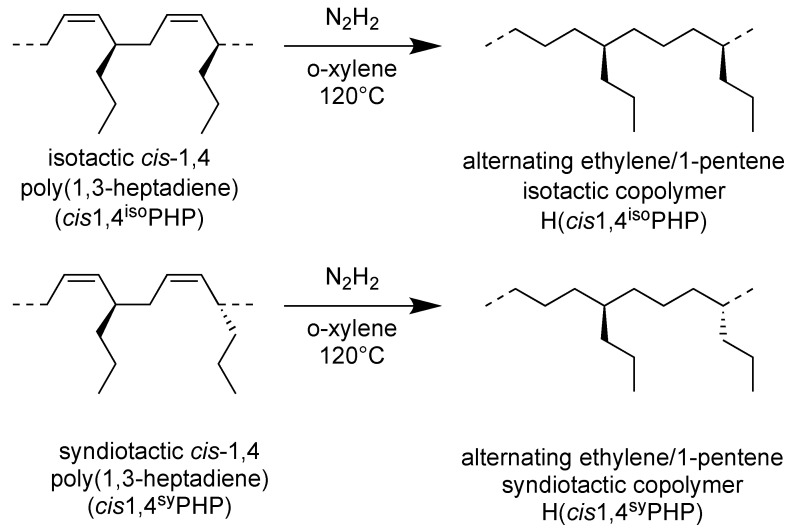
Scheme of hydrogenation of (*cis*1,4^iso^PHP) and (*cis*1,4^sy^PHP) to perfectly alternating isotactic and syndiotactic polypentenes (H(*cis*1,4^iso^PHP) and H(*cis*1,4^sy^PHP)).

The hydrogenation of the isotactic and syndiotactic *cis*-1,4 poly(1,3-heptadiene)s was shown in Scheme 3.

**Figure 3:**
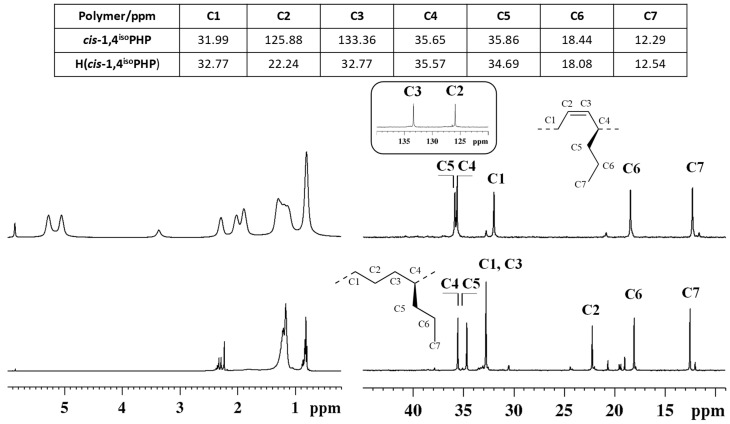
^1^H (**left**) and ^13^C NMR spectra (**right**) of isotactic *cis*-1,4 poly(1,3-heptadiene) (*cis*-1,4^iso^PHP) (**top**) and its saturated E/1-P copolymer (H(*cis*1,4^iso^PHP)) (**bottom**).

**Figure 4:**
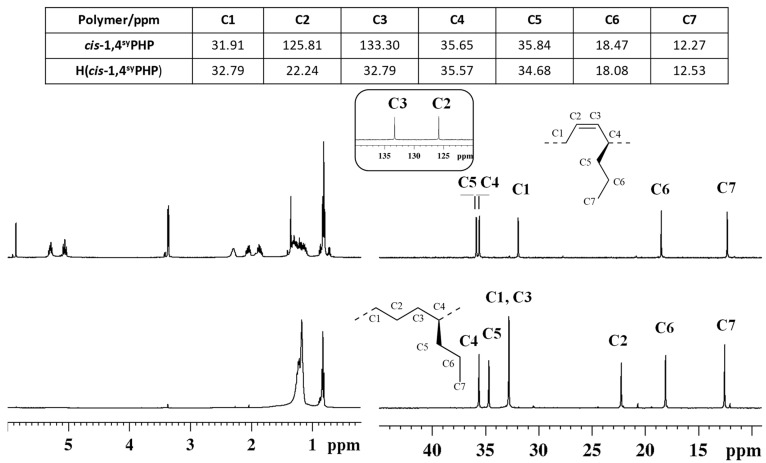
^1^H (**left**) and ^13^C NMR (**right**) spectra of syndiotactic *cis*-1,4 poly(1,3-heptadiene) (*cis*1,4^sy^PHP) (**top**) and its saturated E/1-P copolymer (H(*cis*1,4^sy^PHP)) (**bottom**).

To verify the successful hydrogenation of the diene polymers, FT-IR and ^1^H and ^13^C NMR spectra (Figure 3 and Figure 4) of the starting *cis*-1,4 poly(1,3-diene)s were acquired and compared to the corresponding hydrogenated products.

The peaks attribution of the isotactic and syndiotactic *cis*-1,4 poly(1,3-heptadiene)s and their saturated E/1-P isotactic and syndiotactic copolymers, are shown in Figure 3 and Figure 4, respectively.

NMR data confirmed the complete hydrogenation reaction for both isotactic and syndiotactic *cis*-1,4 poly(1,3-heptadiene)s. Furthermore, the structure and tacticity of the resulting ethylene 1-pentene (E/1P) copolymers were analyzed and assigned through two-dimensional NMR experiments reported in Figures S5–S8. 

As for the stereoregular E/1-B copolymer, the DSC thermograms and the X-ray diffraction of E/1-P copolymers indicate the both iso- and syndiotactc E/1-P copolymers are amorphous with a *T*_g_ of −65 and −63 °C, respectively (Table 2).

### 2.3. Iso- and Syndiotactic Alternating Ethylene/1-Hexene Copolymers (H(cis1,4^iso^PO) and H(cis1,4syndioPO))

**Scheme 4:**
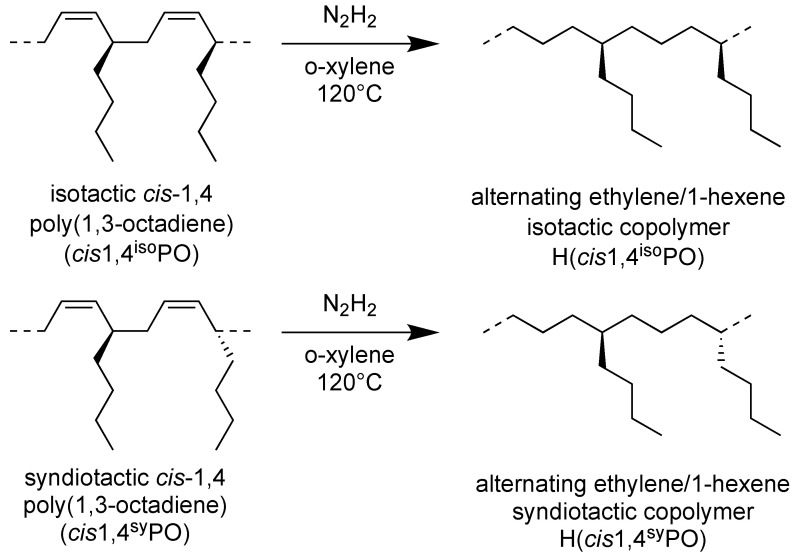
Scheme of hydrogenation of isotactic *cis*-1,4 poly(1,3-octadiene) (*cis*1,4^iso^PO) and syndiotactic *cis*-1,4 poly(1,3-octadiene) (*cis*1,4^sy^PO) to perfectly alternating ethylene-*alt*-1-hexene copolymers, isotactic and syndiotactic (H(*cis*1,4^iso^PO) and H(*cis*1,4^sy^PO)).

**Figure 5:**
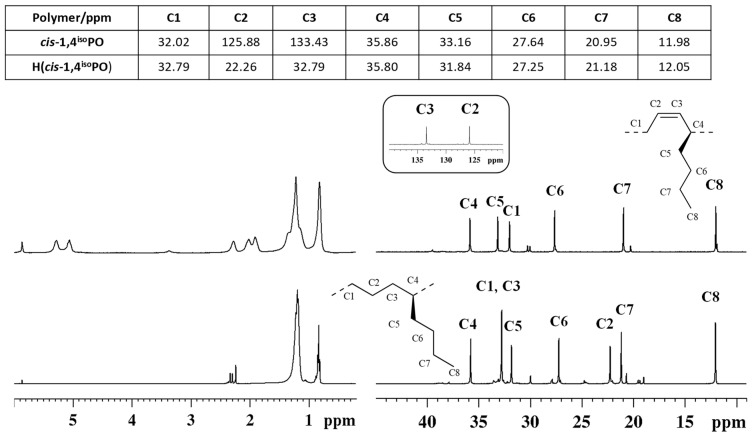
^1^H (**left**) and ^13^C NMR (**right**) spectra of isotactic *cis*-1,4 poly(1,3-octadiene) (*cis*1,4^iso^PO) (**top**) and its saturated E/1-H isotactic copolymer (H(*cis*1,4^iso^PO)) (**bottom**).

**Figure 6:**
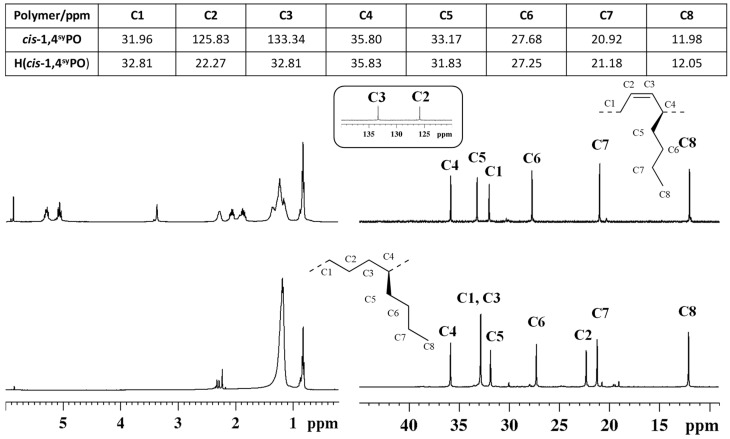
^1^H (**left**) and ^13^C NMR (**right**) spectra of syndiotactic *cis*-1,4 poly(1,3-octadiene) (*cis*1,4^sy^PO) (**top**) and its saturated E/1-H syndiotactic copolymer (H(*cis*1,4^sy^PO)) (**bottom**).

The successful complete hydrogenation of the isotactic and syndiotactic *cis*-1,4 poly(1,3-octadiene)s to alternating E/1-H copolymers, as reported in Scheme 4, was confirmed by FT-IR (Figure S2) and by ^1^H and ^13^C NMR spectra of the starting *cis*-1,4 poly(1,3-diene)s and of the corresponding hydrogenated products (Figure 5 and Figure 6).

The peaks in the olefinic regions due to the olefinic hydrogen atoms (from 5.0 to 5.6 ppm) observed in the ^1^H NMR spectra (Figure 5 and Figure 6) and of olefinic carbon atoms observed in the carbon spectra (at ≈126 and 133 ppm) of the diene polymers are not observed in the spectra of the hydrogenated polymers (Figure 5 and Figure 6), thus confirming indeed the complete hydrogenation reaction.

Also in that case, the polymers microstructure was determined by two dimensional NMR experiments, reported in Figures S9 and S10.

**Figure 7:**
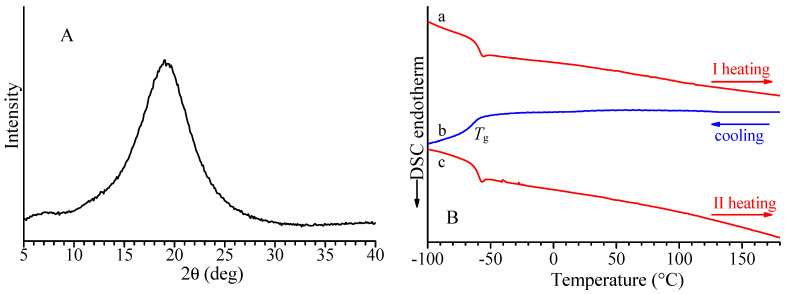
X-ray powder diffraction profile (**A**) and DSC curves (**B**) recorded at 10 °C/min during first heating (a), successive cooling (b), and second heating (c) of alternating E/1-H isotactic copolymer (sample H(*cis*1,4^iso^PO)).

The X-ray diffraction profile of the alternating isotactic ethylene/1-hexene copolymer is shown in Figure 7A, and the DSC curves recorded during the first heating, successive cooling from the melt, and second heating of the melt crystallized samples are reported in Figure 7B. Both X-ray diffraction and DSC data indicate the E/1-H isotactic copolymers are amorphous and are not able to crystallize upon slow cooling from high temperatures or annealing. Similar behavior has been observed for the syndiotactic E/1-H copolymer. The DSC curves show that for both isotactic and syndiotactic E/1-H copolymers, only glass transition temperatures are nearly −60 °C (Figure 7B and Table 2).

## 3. Materials and Methods

### 3.1. General Procedures and Materials

Manipulations of air- and/or moisture-sensitive materials were carried out under an inert atmosphere using a dual vacuum/nitrogen line and standard Schlenk-line techniques. Toluene (Sigma-Aldrich, Milano, MI, Italy, ≥99.7% pure) and heptane (Sigma-Aldrich, ≥99% pure) were refluxed over Na for about 8 h and then distilled and stored over molecular sieves under nitrogen. *o*-Xylene (Sigma-Aldrich, anhydrous grade), *p*-toluenesulfonylhydrazide (TSH, Sigma-Aldrich), and deuterated solvent for NMR measurements (C_2_D_2_Cl_4_) (Cambridge Isotope Laboratories, Inc., Tewksbury, MA, USA) were used as purchased. 1,3-Hexadiene (Aldrich, 99% pure, mixture of (E) and (Z) isomers), 1,3-heptadiene (Chemsampco, Dallas, TX, USA, 99% pure; predominantly E isomer), and 1,3-octadiene (Chemsampco, 99% pure; predominantly E isomer), were refluxed over calcium hydride for about 4 h, then distilled trap-to-trap, and stored under dry nitrogen. Methylaluminoxane (MAO) (10 wt. % solution in toluene, Sigma-Aldrich) was used as received. Diethylaluminum chloride (AlEt_2_Cl) (Aldrich, 97% pure), triisobutylaluminum (Al(*i*Bu)_3_) (Aldrich, 98% pure), and Nd(OCOC_7_H_15_)_3_ (Strem Chemicals, Newburyport, MA, USA) were used as received. CoCl_2_(P^t^Bu_2_Me)_2_ was prepared as described in ref. [38]. Isotactic *cis*-1,4-poly(E-1,3-hexadiene) (*cis*1,4^iso^PHX) [12], isotactic *cis*-1,4-poly(E-1,3-heptadiene) (*cis*1,4i^so^PHP) [13], isotactic *cis*-1,4-poly(E-1,3-octadiene) (*cis*1,4^iso^PO) [13], syndiotactic *cis*-1,4-poly(E-1,3-heptadiene) (*cis*1,4^sy^PHP) [13], and syndiotactic *cis*-1,4-poly(E-1,3-octadiene) (*cis*1,4^sy^PO) [13], were synthesized as reported in the literature.

### 3.2. Hydrogenation Procedure

The hydrogenation was carried out in a round-bottom flask equipped with a magnetic stirring, reflux condenser, nitrogen inlet port, and temperature controller. Typically, the specified amount of the diene polymer was dissolved in *o*-xylene. The reaction mixture was continuously stirred at room temperature until the polymer was completely dissolved. TSH was then added, and the mixture was refluxed by slowly heating to 120 °C. After 3 days, the mixture was allowed to cool spontaneously to room temperature, and TSH was added. This operation is repeated once again. Upon completion of the reaction, the hydrogenated sample was hot-filtered, the volume of the filtered solution was reduced under vacuum, and the dissolved polymer precipitated with methanol and collected by filtration. The polymer was dried under vacuum at room temperature, and then it was extracted with acetone through a Soxhlet method for 10 h in order to remove any excess TSH and byproducts originating from TSH decomposition. The residual polymer was finally dried under a vacuum, dissolved in toluene, precipitated into methanol, and dried again under a vacuum at room temperature to constant weight.

Specifically, for each polymer sample, the hydrogenation conditions were the following: 

*Cis*1,4^iso^PHX isotactic *cis*-1,4 poly(1,3-hexadiene): polymer 0.54 g; xylene, 80 mL; first addition of TSH, 4.2 g (2.25 × 10^−2^ mol); second and third addition of TSH, 7 g (3.74 × 10^−2^ mol); Yield of hydrogenated polymer H(*cis*1,4^iso^PHX): 0.49 g

*Cis*1,4^iso^PHP isotactic *cis*-1,4 poly(1,3-heptadiene): polymer 0.73 g; xylene, 80 mL; first addition of TSH, 5.3 g (2.84 × 10^−2^ mol); second and third addition of TSH, 9.0 g (4.83 × 10^−2^ mol); Yield of hydrogenated polymer H(*cis*1,4i^so^PHP): 0.70 g

*Cis*1,4^sy^PHP syndiotactic *cis*-1,4 poly(1,3-heptadiene): polymer 0.46 g; xylene, 80 mL; first addition of TSH, 3.5 g (1.88 × 10^−2^ mol); second and third addition of TSH, 7.0 g (3.76 × 10^−2^ mol); Yield of hydrogenated polymer H(*cis*1,4^sy^PHP): 0.30 g

*Cis*1,4^iso^PO isotactic *cis*-1,4 poly(1,3-octadiene): polymer 1.08 g; xilene, 125 mL; first addition of THS, 7.7 g (4.76 × 10^−2^ mol); second and third addition of TSH, 7.7 g (4.76 × 10^−2^ mol); Yield of hydrogenated polymer H(*cis*1,4^iso^PO): 0.96 g

*Cis*1,4^sy^PO syndiotactic *cis*-1,4 poly(1,3-octadiene): polymer 0.61 g; xylene, 80 mL; first addition of TSH, 4.3 g (2.3 × 10^−2^ mol); second and third addition of TSH, 9.0 g (4.83 × 10^−2^ mol); Yield of hydrogenated polymer H(*cis*1,4^sy^PO): 0.41 g.

### 3.3. Polymer Characterization

^1^H and ^13^C NMR measurements were carried out on a Bruker (Billerica, MA, USA) Avance 400 spectrometer. The spectra were obtained in C_2_D_2_Cl_4_ at 103 °C (hexamethyldisiloxane, HMDS, as internal standard). The concentration of polymer solutions was about 10 wt %. ^13^C parameters were: spectral width 17 kHz; 90° pulse 11.0 μs PL1 −5.0 dB, with a delay of 16 s. 

The molecular weight averages (*M_w_*) and the molecular weight distribution (*M_w_*/*M_n_*) were obtained by a high-temperature Waters (Milford, MA, USA) GPCV2000 size exclusion chromatography (SEC) system using two online detectors: a differential viscometer and a refractometer. The experimental conditions consisted of three PL Gel Olexis columns, o-DCB as the mobile phase, 0.8 mL min^−1^ flow rate, and 145 °C temperature. Universal calibration of the SEC system was performed using eighteen narrow *M_w_*/*M_n_* polystyrene standards with molar weights ranging from 162 to 5.6 × 10^6^ g mol^−1^. For the analysis, about 12 mg of the polymer was dissolved in 5 mL of *o*-DCB with 0.05% of BHT as the antioxidant.

X-ray powder diffraction profiles were obtained with Ni-filtered Cu Kα radiation by using an Empyrean diffractometer by Malvern Panalytical (Malvern, UK) operating in the reflection geometry with continuous scans of the 2θ angle and scanning rate of 0.02 degree/s

Thermal analysis was performed with a differential scanning calorimeter Mettler-DSC30/2285 (Zurich, Switzerland), equipped with a liquid nitrogen cooling system for measurements at low temperature. The scans were recorded in flowing nitrogen atmosphere at heating or cooling rates of 10 °C/min.

## 4. Conclusions

Several perfectly alternating ethylene/α-olefin copolymers were prepared by hydrogenation of highly stereoregular *cis*-1,4 poly(1,3-diene)s. Most of these polymers were completely new, being hardly obtainable by simple stereospecific polymerization of the corresponding α-olefins. All the polymers were fully characterized to determine their (micro)structural and thermal properties. 

Some of the polymers obtained exhibited interesting properties for possible application as flexible thermoplastic and elastomeric materials thanks to the very low glass transition temperatures; however, most of the monomers used for the preparation of the stereoregular poly(1,3-diene)s are quite expensive, they will hardly be able to find an industrial application, or, in any case, their possible use should be carefully assessed. However, their use as model polymers, useful for evaluating similar polymers, remains extremely significant.

## Figures and Tables

**Table 1:** Polymerization of 1,3-dienes to *cis*-1,4 polymers with neodymium- and cobalt-based catalysts.

Polymer	Mon.	Solvent	Catalyst	Time	Yield	*cis*-1,4 ^a^	*T_g_* ^b^	*M_w_* ^c^	*M_w_*/*M_n_* ^c^
				(h)	(%)	(%)	(°C)	(kg/mol)	
*cis*1,4^iso^*PHX*	HX	heptane	AlEt_2_Cl/Nd(OCOC_7_H_15_)_3_/Al^i^Bu_3_ (3:1:30)	1	87	≥95	−56	170	3.8
*cis*1,4^iso^PHP	HP	heptane	AlEt_2_Cl/Nd(OCOC_7_H_15_)_3_/Al^i^Bu_3_ (3:1:30)	2	74	≥95	−47	120	8.5
*cis*1,4^sy^PHP	HP	toluene	CoCl_2_(P^t^BuMe_2_)_2_/MAO (1:1000)	24	73	≥95	−50	30	1.6
*cis*1,4^iso^PO	O	heptane	AlEt_2_Cl/Nd(OCOC_7_H_15_)_3_/Al^i^Bu_3_ (3:1:30)	2	75	≥99	−52	110	10
*cis*1,4^sy^PO	O	toluene	CoCl_2_(P^t^BuMe_2_)_2_/MAO (1:1000)	24	66	≥94	−53	25	1.8

Polymerization conditions: monomer, 2 mL; solvent, total volume 16 mL; Mt, 2 × 10^−5^ mol; 22 °C. ^a^ Percentage of *cis*-1,4 units, determined by NMR analysis; ^b^ glass transition temperature, determined by DSC; ^c^ determined by SEC. HX = 1,3-hexadiene; HP = 1,3-heptadiene; O = 1,3-octadiene.

**Table 2:** Ethylene-*alt*-*α*-olefin copolymers feature.

Polymer	*M_w_ *^a^ (kg/mol)	*M_w_*/*M_n_* ^a^	*T_g_* ^b^ (°C)
H(*cis*1,4^iso^PHX) isotactic ethylene-*alt*-1-butene copolymer	130	3.5	−66
H(*cis*1,4^iso^PHP) isotactic ethylene-*alt*-1-pentene copolymer	88	6.7	−65
H(*cis*1,4^sy^PHP) syndiotactic ethylene-*alt*-1-pentene copolymer	28	1.5	−63
H(*cis*1,4^iso^PO) isotactic ethylene-*alt*-1-hexene copolymer	82	7.2	−60
H(*cis*1,4^sy^PO) syndiotactic ethylene-*alt*-1-hexene copolymer	24	1.7	−60

^a^ Determined by SEC; ^b^ determined from the DSC cooling thermograms.

## Data Availability

Data are contained within the article and Supplementary Materials.

## Supplementary Materials

The following supporting information can be downloaded at: https://www.mdpi.com/article/10.3390/molecules29061376/s1. FT-IR spectra of the diene polymers and ethylene-*alt*-α-olefin copolymers; two dimensional NMR spectra of the ethylene-*alt*-α-olefin copolymers. Figure S1. FTIR spectra of isotactic *cis*-1,4 poly(1,3-hexadiene) (top, (*cis*1,4^iso^PHX)) and its saturated E/1-B copolymer polymer (bottom, H(*cis*1,4^iso^PHX); Figure S2. FTIR spectra of (A) isotactic *cis*-1,4 poly(1,3-octadiene) (*cis*1,4^iso^PO) (top) and its saturated E/1-H isotactic copolymer (H(*cis*1,4^iso^PO)) (bottom), (B) syndiotactic *cis*-1,4 poly(1,3-octadiene) (*cis*1,4^sy^PO) (top) and its saturated E/1-H syndiotactic copolymer (H(*cis*1,4^sy^PO)) (bottom); Figure S3. Two dimensional ^1^H-^13^C HSQC and ^1^H-^13^C HMBC spectra of ethylene-*alt*-1-butene copolymer, H(*cis*1,4^iso^PHX); Figure S4. Two dimensional ^1^H-^1^H COSY and ^1^H-^1^H TOCSY spectra of ethylene-*alt*-1-butene copolymer, H(*cis*1,4^iso^PHX); Figure S5. Two dimensional ^1^H-^13^C HSQC and ^1^H-^13^C HMBC spectra of ethylene-*alt*-1-pentene copolymer, H(*cis*1,4^iso^PHP); Figure S6. Two dimensional ^1^H-^1^H COSY and ^1^H-^1^H TOCSY spectra of ethylene-*alt*-1-pentene copolymer, H(*cis*1,4^iso^PHP); Figure S7. Two dimensional ^1^H-^13^C HSQC and ^1^H-^13^C HMBC spectra of ethylene-*alt*-1-pentene copolymer, H(*cis*1,4^sy^PHP); Figure S8. Two dimensional ^1^H-^1^H COSY and ^1^H-^1^H TOCSY spectra of ethylene-*alt*-1-pentene copolymer, H(*cis*1,4^sy^PHX); Figure S9. Two dimensional ^1^H-^13^C HSQC and ^1^H-^13^C HMBC spectra of ethylene-*alt*-1-hexene copolymer, H(*cis*1,4^iso^PO); Figure S10. Two dimensional ^1^H-^1^H COSY and ^1^H-^1^H TOCSY spectra of ethylene-*alt*-1-hexene copolymer, H(*cis*1,4^iso^PHO).
