# Supplementary material for: Ethylene-alt-α-Olefin Copolymers by Hydrogenation of Highly Stereoregular cis-1,4 Polydienes: Synthesis and Structural Characterization
Source: Molecules. 2024 Mar 20;29(6):1376. doi: 10.3390/molecules29061376 (PMC10974103; doi:10.3390/molecules29061376)
Supplement: Supplementary file 1 [file molecules-29-01376-s001.zip › molecules-2887367-supplementary.pdf]

Article

Ethylene-*alt*-α-Olefin Copolymers by Hydrogenation of Highly Stereoregular *cis*-1,4 Polydienes: Synthesis and Structural Characterization

Giovanni Ricci [1,*], Antonella Caterina Boccia [1], Ivana Pierro [2], Claudio De Rosa [3] and Miriam Scoti [3]

[1] Consiglio Nazionale delle Ricerche (CNR)—Istituto di Scienze e Tecnologie Chimiche "Giulio Natta" (SCITEC), Via A. Corti 12, I-20133 Milano, Italy; antonella.boccia@scitec.cnr.it
[2] Scientific Advisor, I-21052 Busto Arsizio, Italy; ivanapierro@gmail.com
[3] Dipartimento di Scienze Chimiche, Università di Napoli Federico II, Complesso Monte S. Angelo, Via Cintia, I-80126 Napoli, Italy; claudio.derosa@unina.it (C.D.R.); miriam.scoti@unina.it (M.S.)
* Correspondence: giovanni.ricci@scitec.cnr.it

Supplementary materials

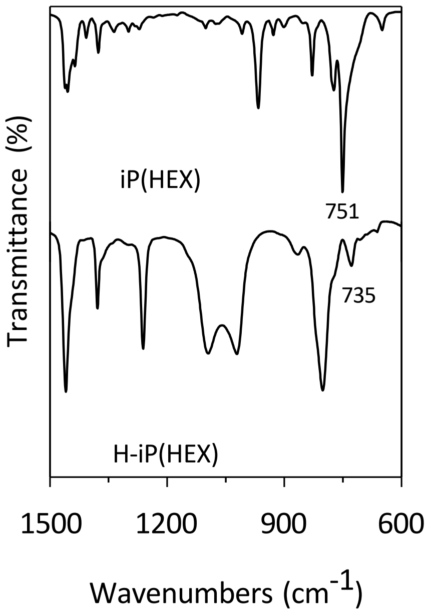

Figure S1. FTIR spectra of isotactic *cis*-1,4 poly(1,3-hexadiene) (top, (***cis*1,4^{iso}PHX**)) and its saturated E/1-B copolymer polymer (bottom, **H(*cis*1,4^{iso}PHX**).

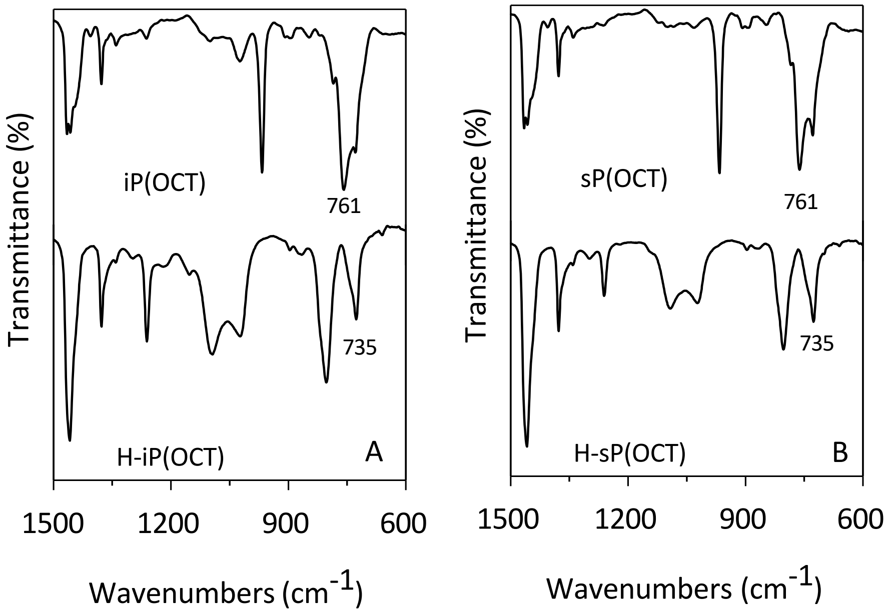

Figure S2. FTIR spectra of (A) isotactic *cis*-1,4 poly(1,3-octadiene) (***cis*1,4^{iso}PO**) (top) and its saturated E/1-H isotactic copolymer (**H(*cis*1,4^{iso}PO))** (bottom), (B) syndiotactic *cis*-1,4 poly(1,3-octadiene) (***cis*1,4^{sy}PO**) (top) and its saturated E/1-H syndiotactic copolymer (**H(*cis*1,4^{sy}PO))** (bottom).

Two-dimensional NMR characterization

Two-dimensional heteronuclear ^1H-^{13}C experiments were acquired on a Bruker AVANCE DMX spectrometer operating at 600 MHz, (14.1 T), and 330 K. The g-HSQC experiment (Gradient-Heteronuclear Single Quantum Correlation) was performed by applying a coupling constant $^1J_{CH}$ = 125 Hz; data matrix 2K × 256; number of scans:128; P1 as 90° pulse was determined on each sample. The g-HMBC experiments (gradient-Heteronuclear Multiple Bond Correlation) were performed by applying a delay of 100 ms for the evolution of long-range coupling; data matrix 2K × 512; number of scans 64; D1 2.00 s. Data were zero-filled and weighted with a sine bell function before Fourier transformation. COSY experiments: data matrix 1K × 512; number of scans 32; D1 2.00 s. TOCSY experiments: data matrix 1K × 512; number of scans 32; D1 2.00 s; tocsy mixing time 90 ms. Differences in chemical shifts among mono- and two-dimensional experiments are due to the different temperatures of data acquisition. Differences in chemical shifts among mono- and two-dimensional experiments are due to the different temperatures of data acquisition.

^1H-^{13}C HSQC (heteronuclear single quantum coherence) NMR experiment is used to determine proton-carbon single bond correlations.

^1H-^{13}C HMBC (heteronuclear multiple bond correlation) NMR experiment gives correlations between carbons and protons that are two and three bonds away.

^1H-^1H COSY experiment (homonuclear Correlation Spectroscopy) is used to identify spins that are coupled to each other.

^1H-^1H TOCSY experiment (homonuclear Total Correlation Spectroscopy) is used to identify correlations between all protons within a given spin system.

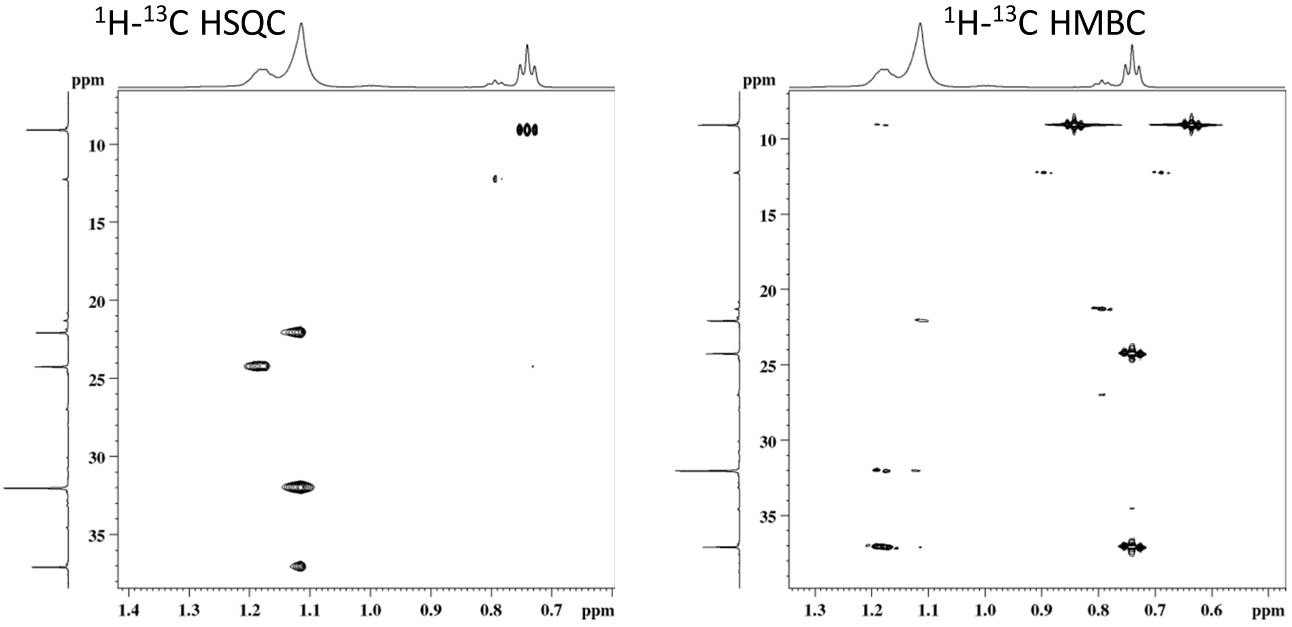

Figure S3: Two dimensional ^1H-^{13}C HSQC and ^1H-^{13}C HMBC spectra of the ethylene-*alt*-1-butene copolymer, **H(cis1,4^{iso}PHX).**

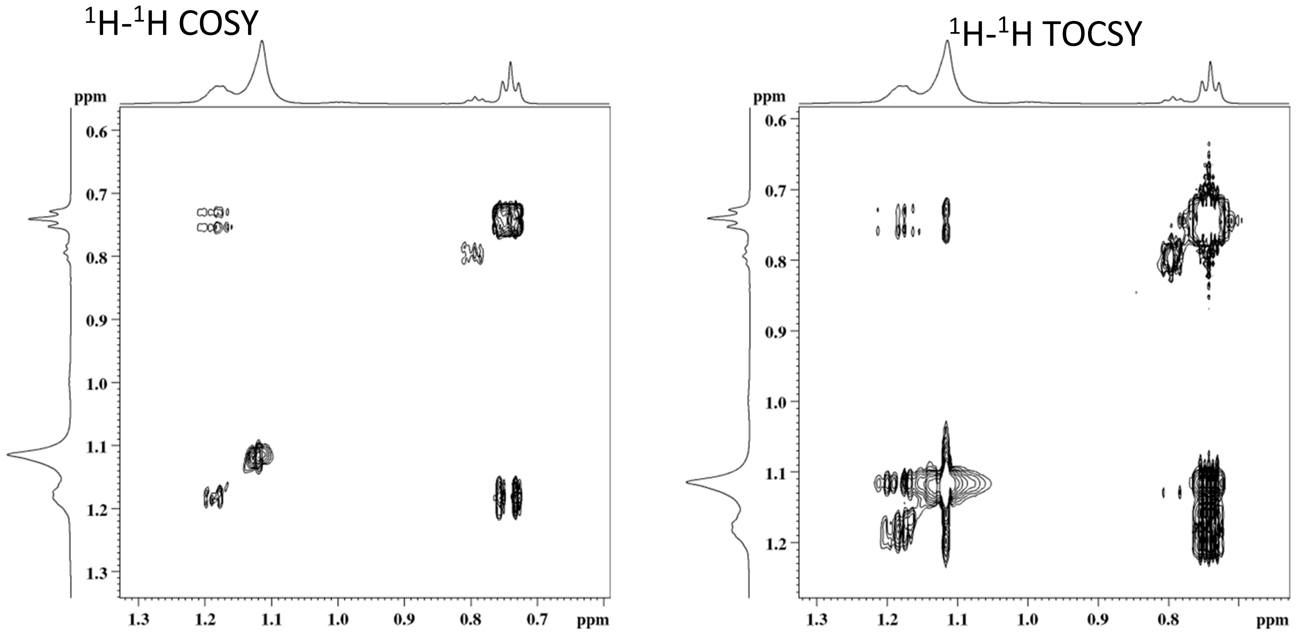

Figure S4: Two dimensional ^{1}H-^{1}H COSY and ^{1}H-^{1}H TOCSY spectra of the ethylene-*alt*-1-butene copolymer, **H(cis1,4^{iso}PHX).**

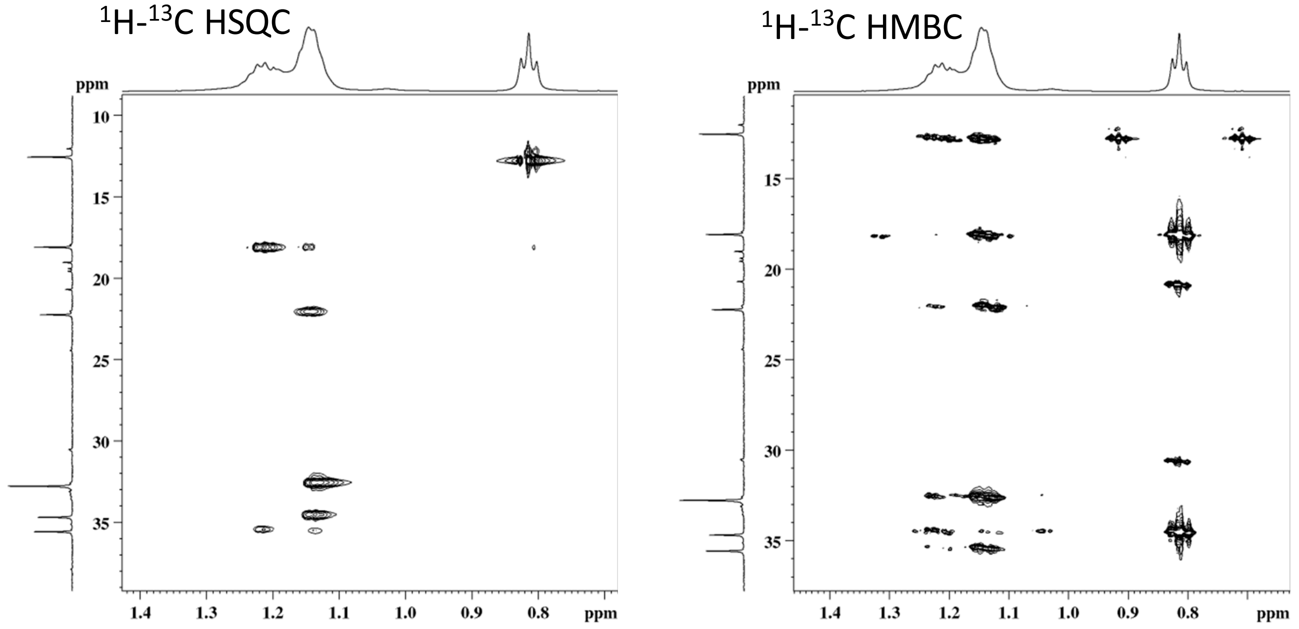

Figure S5: Two dimensional ^{1}H-^{13}C HSQC and ^{1}H-^{13}C HMBC spectra of ethylene-*alt*-1-pentene copolymer, **H(cis1,4^{iso}PHP).**

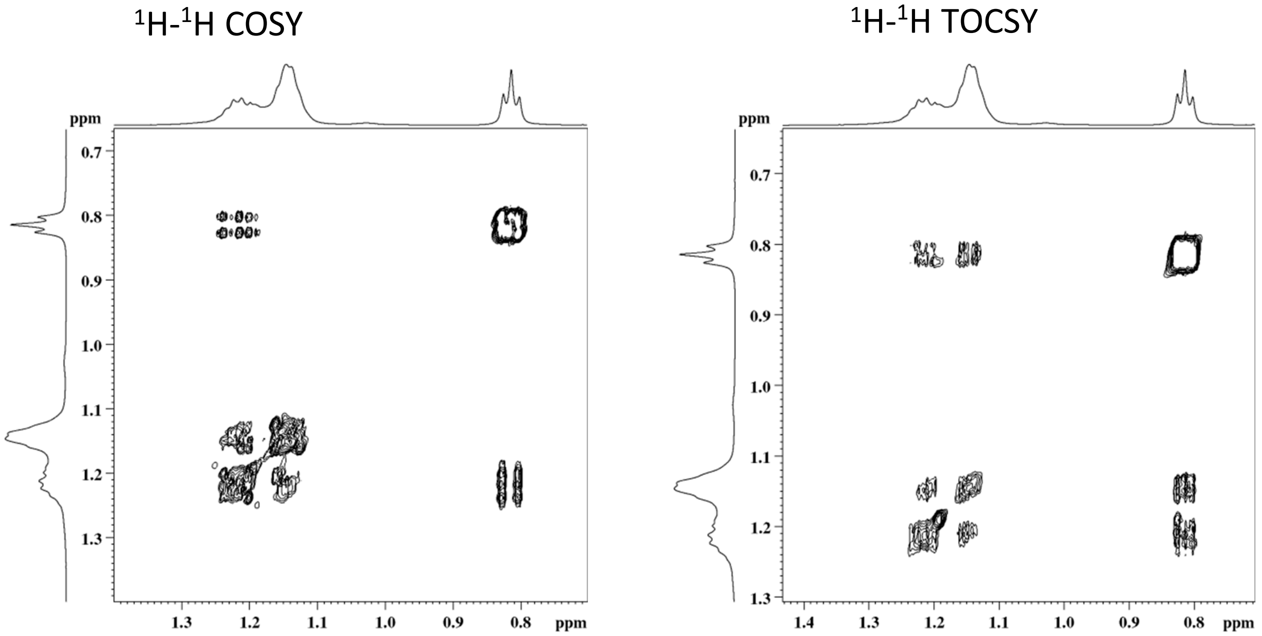

Figure S6: Two dimensional ^{1}H-^{1}H COSY and ^{1}H-^{1}H TOCSY spectra of the ethylene-*alt*-1-pentene copolymer, **H(cis1,4^{iso}PHP).**

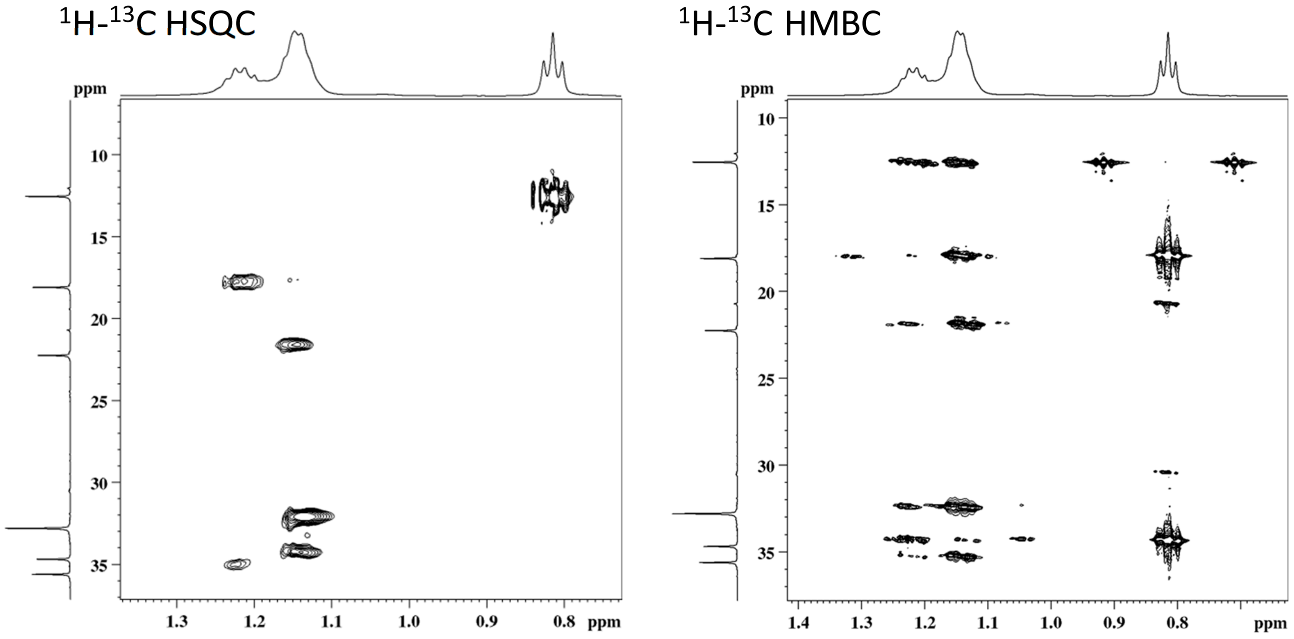

Figure S7: Two dimensional ^{1}H-^{13}C HSQC and ^{1}H-^{13}C HMBC spectra of the ethylene-*alt*-1-pentene copolymer, **H(cis1,4^{sy}PHP).**

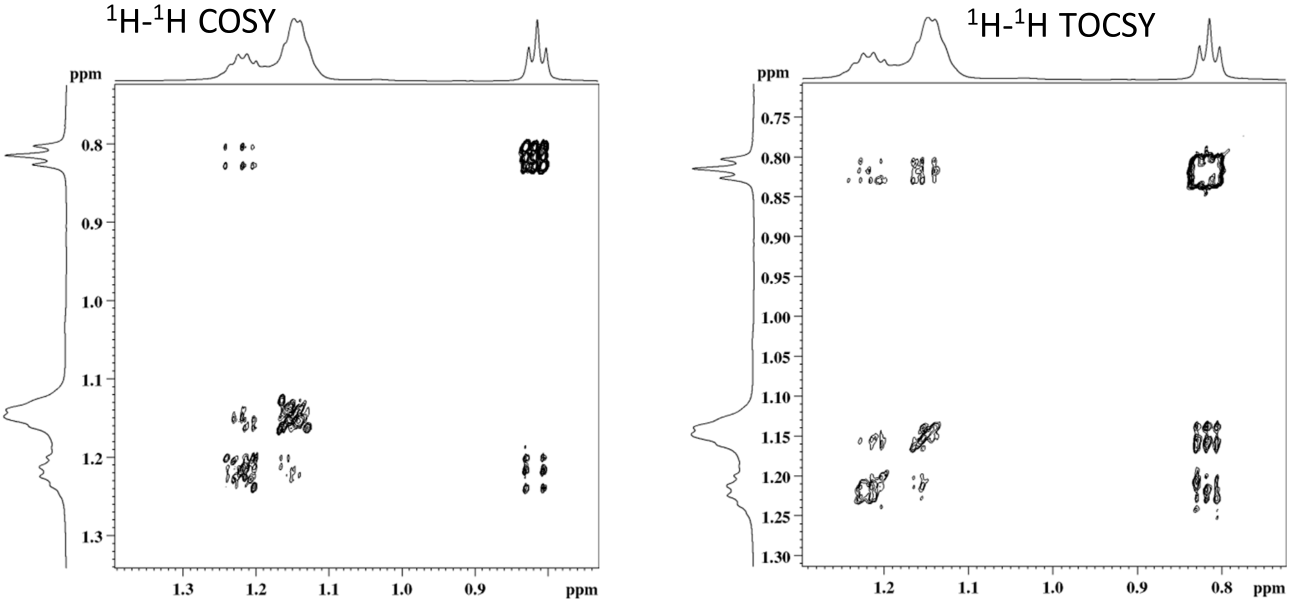

Figure S8: Two dimensional ^{1}H-^{1}H COSY and ^{1}H-^{1}H TOCSY spectra of the ethylene-*alt*-1-pentene copolymer, **H(cis1,4^{sy}PHX).**

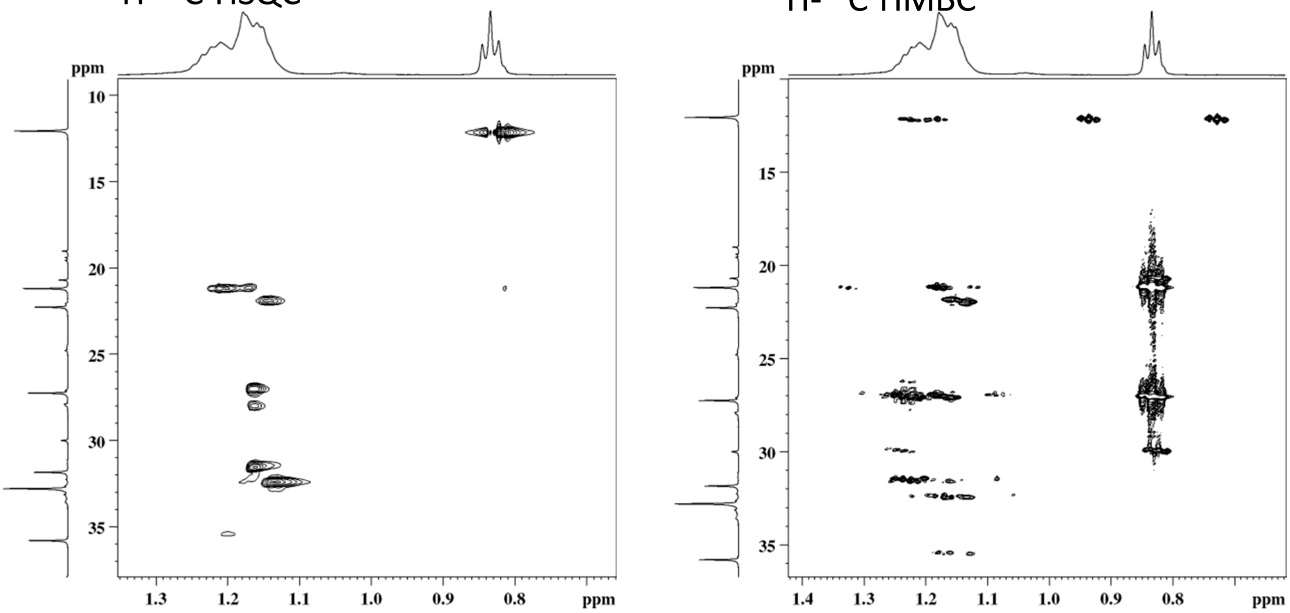

Figure S9: Two dimensional ^{1}H-^{13}C HSQC and ^{1}H-^{13}C HMBC spectra of ethylene-*alt*-1-hexene copolymer, **H(cis1,4^{iso}PO).**

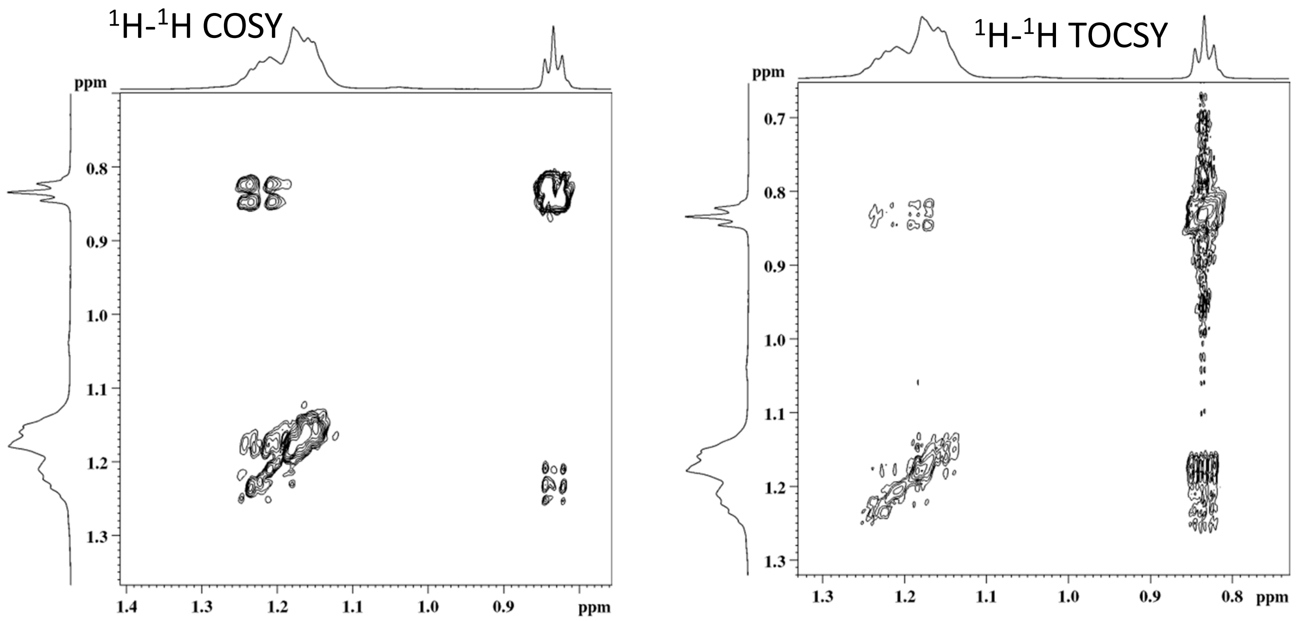

Figure S10: Two dimensional ^{1}H-^{1}H COSY and ^{1}H-^{1}H TOCSY spectra of the ethylene-*alt*-1-hexene copolymer, **H(cis1,4^{iso}PHO).**
